# Application of susceptibility-weighted imaging in staging diagnosis of ischemic cerebral infarction and its prognostic value

**DOI:** 10.3389/fneur.2026.1722009

**Published:** 2026-02-23

**Authors:** Dexing Zhou, He Lu, Jin Duan, Jiali Wang, He Zhang, Cong Song

**Affiliations:** 1 Department of Radiology, The People's Hospital of Jiawang District of Xuzhou, Xuzhou, Jiangsu, China; 2 Department of Radiology, Shanghai Pulmonary Hospital, School of Medicine, Tongji University, Shanghai, China; 3 Department of Radiology, The Affiliated Hospital of Xuzhou Medical University, Xuzhou, Jiangsu, China

**Keywords:** ischemic cerebral infarction, neurological function, prognosis, prognostic value, susceptibility-weighted imaging

## Abstract

**Objective:**

This study aims to evaluate the imaging characteristics of susceptibility-weighted imaging (SWI) in patients with ischemic cerebral infarction at different stages and to investigate its association with neurological outcomes and prognostic value.

**Methods:**

A total of 165 patients with ischemic cerebral infarction were enrolled, including 97 in the acute phase and 68 in the recovery phase. All patients underwent 3.0 Tesla (3.0T) SWI examination. The presence of prominent vessel sign (PVS), susceptibility vessel sign (SVS), hemorrhagic transformation (HI), and cerebral microbleeds (CMBs) was recorded. National Institutes of Health Stroke Scale (NIHSS), modified Rankin Scale (mRS), and Barthel Index, were compared between groups. Spearman correlation analysis and receiver operating characteristic (ROC) curve analysis were used to assess the relationship between SWI indicators and prognosis.

**Results:**

The recovery-phase group showed significantly higher rates of PVS positivity and HI positivity, as well as a greater number of CMBs. Patients in the acute-phase group had higher NIHSS scores at both admission and discharge, higher 3-month mRS scores, and lower Barthel Index scores. Correlation analysis revealed that PVS, SVS, HI, and CMBs were positively correlated with NIHSS and mRS scores but negatively correlated with the Barthel Index, with CMBs showing the strongest association (*r* = −0.855, *p* < 0.001). ROC curve analysis indicated that CMBs had the best predictive performance for poor prognosis, outperforming PVS, SVS, and, HI.

**Conclusion:**

SWI can reveal imaging differences across different stages of ischemic cerebral infarction and its indicators are closely associated with neurological dysfunction.

## 1 Introduction

Globally, ischemic stroke (IS) ranks among the foremost contributors to mortality and long-term disability, with both incidence and recurrence rates continuing to rise, posing a serious threat to human health and imposing a heavy socioeconomic burden (1, 2). Clinical prognostic assessment plays a critical role in guiding treatment decisions, formulating rehabilitation plans, and predicting long-term outcomes (3). However, traditional neurological function rating scales, such as the National Institutes of Health Stroke Scale (NIHSS), the modified Rankin Scale (mRS), and the Barthel Index, although widely used, primarily rely on clinical symptoms and signs. As a result, they are limited in their ability to objectively and promptly reflect early pathophysiological changes within ischemic lesions (4).

In recent years, magnetic resonance imaging (MRI) has become an essential tool in the diagnosis and management of stroke. Among these techniques, diffusion-weighted imaging (DWI) and magnetic resonance angiography (MRA) are well established for infarct detection and vascular lesion assessment (5). Nevertheless, these imaging modalities remain limited in sensitivity for detecting microbleeds, microcirculatory disturbances, and intravascular thrombi (6). Susceptibility-weighted imaging (SWI), a highly sensitive MRI technique, can simultaneously visualize venous oxygenation changes, thrombus signals, and cerebral microbleeds (CMBs), and is therefore considered to have unique advantages in assessing stroke pathophysiology (7). Previous studies have suggested that SWI-detected markers including the prominent vessel sign (PVS) (8), the susceptibility vessel sign (SVS) (9), hemorrhagic transformation (HI) (10), and the number of CMBs (11), are associated with stroke severity and prognosis. However, systematic investigations of SWI characteristics across different stages of cerebral infarction and their relationship with functional outcomes remain limited (7, 12).

Current research gaps are mainly reflected in the following aspects. First, the distribution patterns of SWI features in acute vs. recovery-phase IS patients have not been fully elucidated. Second, the degree of association between different SWI indicators, neurological dysfunction, and clinical prognosis has not been systematically compared. Third, limited evidence supports the value of SWI in predicting poor outcomes after IS. These limitations restrict the broader clinical application of SWI in risk stratification and precision prognostic assessment. To address these issues, the present study prospectively enrolled patients with acute-phase and recovery-phase IS. By systematically comparing SWI features across different stages and integrating neurological function scales with correlation and receiver operating characteristic (ROC) analyses, we aim to clarify the prognostic role of SWI indicators and to explore their potential value in early clinical evaluation and decision-making.

## 2 Materials and Methods

### 2.1 Baseline characteristics

The sample size was estimated based on previous studies, using the mRS as the primary outcome measure. The calculated effect size was 0.47. At a significance level of α = 0.05 and statistical power of 0.80, the required theoretical sample size was 144 cases. To enhance the robustness of the study, a total of 165 patients were ultimately enrolled.

Participants were consecutive patients diagnosed with ischemic cerebral infarction and admitted to the Department of Neurology of our hospital between January 2023 and December 2024. Diagnosis was confirmed by both clinical and imaging criteria. This study adopted a cross-sectional design, and each patient was enrolled only once and classified into a single phase according to the time from symptom onset at admission. Patients were divided into the acute-phase group ( ≤ 7 days, *n* = 97) and the recovery-phase group (>7 days, *n* = 68) according to the time from symptom onset. The grouping criteria were based on the consensus definitions from the Stroke Recovery and Rehabilitation Roundtable, which define the acute phase as within 7 days after stroke onset, followed by the subacute and recovery phases (13). In the present study, the recovery-phase group primarily included patients in the subacute recovery stage (>7 days after onset), and patients in the chronic phase were not included. Prior to participation, all patients and their families signed informed consent forms, and the study was approved by the hospital's ethics committee.

Inclusion criteria: (1) meeting the diagnostic criteria for ischemic cerebral infarction (14); (2) interval between symptom onset and MRI examination ≤ 72 h at baseline; (3) lesion were restricted to the vascular distribution of a single internal carotid artery or middle cerebral artery.

Exclusion criteria: (1) patients in coma or with severe disease (NIHSS >25 points); (2) previous history of IS; (3) history of intracranial hemorrhage within the past 6 months; (4) contraindications to MRI.

### 2.2 Imaging methods

All patients underwent MRI using a GE Discovery 750 3.0 Tesla (3.0T) scanner (GE Healthcare, United States) equipped with a 20-channel head-and-neck coil. Patients were positioned supine, and the head was immobilized to minimize motion artifacts. Routine sequences included T1-weighted imaging (T1WI), T2-weighted imaging (T2WI), DWI, and three-dimensional time-of-flight MRA (3D-TOF MRA), which were used to assess infarct extent, core regions, and responsible vessels. SWI was performed using a gradient echo sequence with the following parameters: repetition time (TR) = 37.4 ms, echo time (TE) = 22.9 ms, slice thickness = 2 mm, interslice gap = 0 mm, number of slices = 64, matrix = 416 × 320, number of excitations (NEX) = 0.70, bandwidth = 62.5 kHz, and flip angle = 20 °. Raw data were transferred to the GE AW4.6 workstation, reconstructed into phase and magnitude images, and processed with maximum intensity projection (MIP).

The images were independently evaluated by two attending radiologists, each possessing five or more years of clinical experience. In cases of disagreement, the final decision was made by an associate chief radiologist. SWI evaluation included the following: (1) PVS: defined as markedly thickened, tortuous, or hypointense venous structures surrounding the infarct core. (2) SVS: defined as punctate or linear hypointense signals within the responsible arterial territory. (3) HI: defined as punctate or patchy hypointense signals within the infarct region. (4) CMBs: defined as small, round or ovoid hypointense lesions on SWI, typically ≤ 10 mm in diameter, and located outside the infarct region; the number of lesions was counted and recorded individually.

### 2.3 Observation indicators

**Figure 1:**
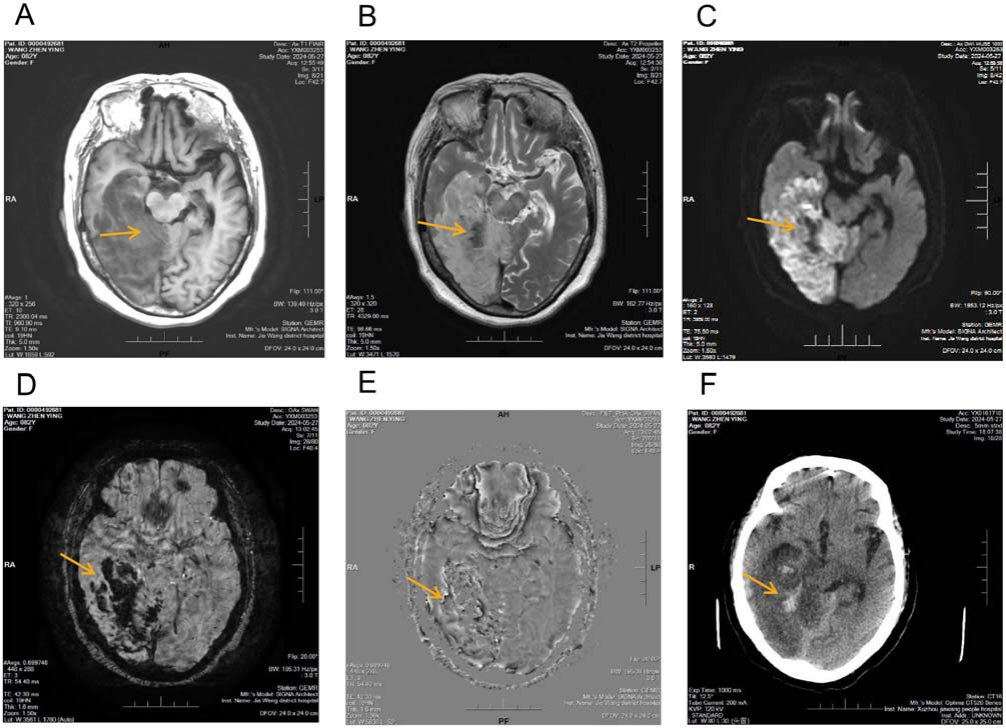
Multimodal imaging findings of acute cerebral infarction with hemorrhagic transformation involving the right frontal, temporal, parietal, and occipital lobes. **(A)** T1-weighted image demonstrating a large hypointense area corresponding to the infarcted region, without definite identification of hemorrhagic components; **(B)** T2-weighted image demonstrating extensive hyperintensity consistent with infarction, with internal focal hypointense areas of nonspecific appearance; **(C)** Diffusion-weighted image demonstrating extensive hyper intensity in the corresponding region, consistent with acute ischemic infarction (yellow arrows); **(D)** SWI magnitude image showing multiple irregular patchy hypo intense foci within the infarcted area (yellow arrows); **(E)** Corresponding SWI phase image demonstrating phase alterations at the same locations (yellow arrows); **(F)** Non-contrast CT showing subtle patchy hyperdensities in the affected region (yellow arrows).

**Figure 2:**
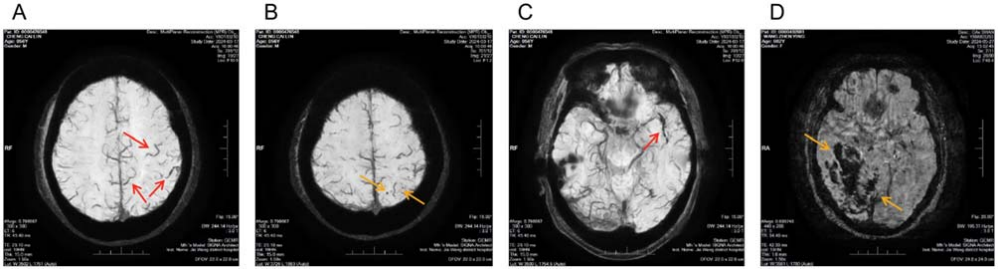
Representative susceptibility-weighted imaging findings in ischemic cerebral infarction from different patients. **(A)** Prominent vessel sign (PVS): SWI magnitude image showing increased number and caliber of cortical and subcortical veins adjacent to the infarcted region (red arrows); **(B)** Cerebral microbleeds (CMBs): SWI magnitude image demonstrating scattered punctate hypointense lesions within the brain parenchyma (yellow arrows); **(C)** Susceptibility vessel sign (SVS): SWI magnitude image showing a linear hypointense signal within the responsible artery (red arrows); **(D)** Hemorrhagic transformation (HI): SWI magnitude image demonstrating irregular patchy hypointense foci within the infarcted area (yellow arrows).

Neurological function was assessed using the NIHSS at admission and discharge, which ranges from 0 to 42, with higher scores indicating more severe neurological deficits (15). At 3 months post-discharge, patients were followed up for the modified Rankin Scale (mRS) (range 0–6, with higher scores indicating greater disability) and Barthel Index scores (range 0–100, with higher scores reflecting better performance in activities of daily living) (16). A good prognosis was defined as mRS ≤ 2 (17). All clinical assessments were independently performed by experienced neurologists with more than 5 years of clinical experience in stroke management, who had received standardized training in the use of these scales. Brain MRI examinations, including SWI, were performed only at baseline after admission, with no follow-up MRI scans obtained during the follow-up period. Hemorrhagic transformation was determined based on SWI findings acquired at baseline. Representative SWI magnitude and phase images are presented in Figures 1, 2.

### 2.4 Statistical analysis

All statistical analyses were performed using SPSS version 26.0 (IBM Corp., Armonk, NY, United States). Normally distributed continuous variables were expressed as mean ± standard deviation ($\bar{x}$ ± s) and compared using the independent-samples *t*-test. Non-normally distributed continuous variables were expressed as median (P25, P75) and compared using the Mann–Whitney *U* test. Categorical variables were expressed as *n* (%) and compared using the χ^2^ test. Correlation analysis was performed using Spearman's rank correlation coefficient. ROC curve analysis was used to evaluate the predictive performance of SWI indicators for poor prognosis. A *P* value < 0.05 was considered statistically significant.

## 3 Results

### 3.1 Baseline characteristics

There were no significant differences between the two groups in age, sex, body mass index (BMI), time from onset to admission, or ength of hospital stay (all *p* > 0.05; Table 1).

**Table 1:** Comparison of baseline characteristics between the two groups [x̄ ± s, *n* (%)].

**Variables**		**Acute phase group (*n* = 97)**	**Recovery phase group (*n* = 68)**	***t/x^2^***	***P***
Age (year)		52.52 ± 5.79	53.85 ± 5.87	1.45	0.15
Sex	Male	58 (56.86%)	44 (61.9%)	0.41	0.52
	Female	39 (43.14%)	24 (38.1%)		
BMI (kg/m^2^)		23.54 ± 1.58	23.29 ± 1.69	0.98	0.33
Time from recent symptom aggravation to admission (h)		17.55 ± 3.42	17.23 ± 3.37	0.61	0.54
Length of hospital stay (days)		6.17 ± 1.07	6.51 ± 1.38	1.80	0.08

### 3.2 Distribution of SWI features in patients at different stages

The distribution of SWI features varied across stages. The positive rate of the PVS was significantly higher in the recovery-phase group than in the acute phase group [42.65% (29/68) vs. 26.80% (26/97); *z* = 4.52, *P* = 0.03]. The positive rate of HI was also higher in the recovery-phase group than in the acute phase group [25.00% (17/68) vs. 10.31% (10/97); *z* = 6.30, *P* = 0.01]. In addition, the median number of CMBs was significantly greater in the recovery phase group compared with the acute phase group [3 (2, 4) vs. 1 (0, 2); *z* = 7.88, *P* < 0.001]. By contrast, no significant difference in the positive rate of the SVS was observed between the two groups (*P* = 0.53) (Table 2).

**Table 2:** Distribution of SWI features in patients at different stages.

**Group**	**PVS positivity**	**SVS positivity**	**HI positivity**	**CMBs**
Acute phase group (*n* = 97)	26 (26.8%)	24 (24.74%)	10 (10.31%)	1 (0, 2)
Recovery phase group (*n* = 68)	29 (42.65%)	14 (20.59%)	17 (25.0%)	3 (2, 4)
*x^2^/z*	4.52	0.39	6.30	7.88
*P*	0.03	0.53	0.01	< 0.001

### 3.3 Comparison of neurological function indicators

Neurological function indicators differed significantly between groups. Compared with the recovery-phase group, patients in the acute-phase group had significantly higher NIHSS scores at admission (*z* = 6.65, *P* < 0.001) and at discharge (*z* = 6.98, *P* < 0.001). At 3 months, the acute-phase group showed higher mRS scores (*z* = 9.41, *P* < 0.001) and lower Barthel Index scores (*t* = 3.97, *P* < 0.001) than the recovery-phase group (Table 3).

**Table 3:** Comparison of neurological function between patients with acute and recovery-stage IS [(P_25_, P_75_), $\bar{x}$ ± s].

**Group**	**NIHSS at admission**	**NIHSS at discharge**	**mRS**	**Barthel**
Acute phase group (*n* = 97)	8 (6.5, 10)	4 (3, 5)	2 (2, 3)	83.51 ± 4.44
Recovery phase group (*n* = 68)	5 (5, 6)	2 (1, 3)	1 (1, 2)	88.10 ± 4.27
*t/z*	6.65	6.98	9.41	3.97
*P*	< 0.001	< 0.001	< 0.001	< 0.001

### 3.4 Correlation between SWI features and neurological function scores

Correlation analysis demonstrated that PVS was positively correlated with admission NIHSS (*r* = 0.527, *P* < 0.001), discharge NIHSS (*r* = 0.439, *P* < 0.001), and mRS (*r* = 0.288, *P* < 0.001), and negatively correlated with the Barthel Index (*r* = −0.744, *P* < 0.001). SVS was positively correlated with admission NIHSS (*r* = 0.462, *P* < 0.001), discharge NIHSS (*r* = 0.385, *P* < 0.001), and mRS (*r* = 0.250, *P* = 0.001), and negatively correlated with the Barthel Index (*r* = −0.672, *P* < 0.001). HI was positively correlated with admission NIHSS (*r* = 0.381, *P* < 0.001), discharge NIHSS (*r* = 0.260, *P* = 0.001), and mRS (*r* = 0.247, *P* < 0.001), and negatively correlated with the Barthel Index (*r* = −0.582, *P* < 0.001). CMBs were strongly positively correlated with admission NIHSS (*r* = 0.671, *P* < 0.001), discharge NIHSS (*r* = 0.530, *P* < 0.001), and mRS (*r* = 0.343, *P* < 0.001), and highly negatively correlated with the Barthel Index (*r* = −0.855, *P* < 0.001). These results are summarized in Table 4.

**Table 4:** Correlation between SWI parameters and clinical scores[*r (P*)].

**SWI indicators**	**NIHSS at admission**		**NIHSS at discharge**		**mRS**		**Barthel**	
	***r***	***P***	***r***	***P***	***r***	***P***	***r***	***P***
PVS	0.527	< 0.001	0.439	< 0.001	0.288	< 0.001	−0.744	< 0.001
SVS	0.462	< 0.001	0.385	< 0.001	0.250	0.001	−0.672	< 0.001
HI	0.381	< 0.001	0.260	0.001	0.247	< 0.001	−0.582	< 0.001
CMBs	0.671	< 0.001	0.530	< 0.001	0.343	< 0.001	−0.855	< 0.001

### 3.5 ROC analysis of SWI indicators for predicting poor prognosis

**Figure 3:**
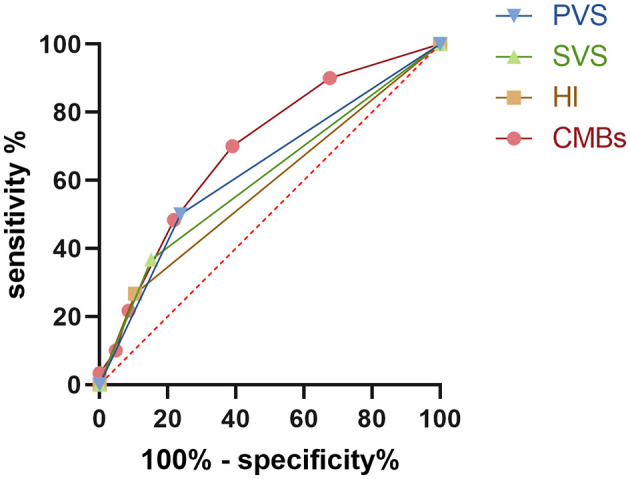
ROC curve analysis of the diagnostic efficacy of SWI indicators in predicting adverse prognosis.

ROC curve analysis indicated that all SWI indicators had predictive value for poor prognosis in IS patients (*P* < 0.05 for all). Among them, the number of CMBs demonstrated the highest predictive performance (AUC = 0.6935, 95% CI: 0.6109–0.7761), with sensitivity of 70%, specificity of 60.95%, and the highest Youden index (0.3095). ROC analysis further identified an optimal cutoff value of ≥2 CMBs for predicting poor prognosis. PVS ranked second (AUC = 0.6310, 95% CI: 0.5406–0.7213; sensitivity 50%, specificity 76.19%; Youden index = 0.2619). For binary SWI markers including PVS, SVS, and HI, a positive finding was considered the optimal cutoff value. The predictive performance of SVS and HI was relatively lower, with AUC values of 0.6071 (95% CI: 0.5148–0.6994) and 0.5810 (95% CI: 0.4878–0.6741), respectively. For SVS, sensitivity was 36.67% and specificity 84.76%; for HI, sensitivity was 26.67% and specificity 89.52% (Table 5 and Figure 3).

**Table 5:** ROC analysis of SWI parameters for predicting unfavorable outcomes.

**Indicators**	**AUC**	**sensitivity**	**specificity**	**Youden index**	**Optimal cutoff value**	**95% CI**
PVS	0.6310	50%	76.19%	0.2619	Positive	0.5406–0.7213
SVS	0.6071	36.67%	84.76%	0.2143	Positive	0.5148–0.6994
HI	0.5810	26.67%	89.52%	0.1619	Positive	0.4878–0.6741
CMBs	0.6935	70%	60.95%	0.3095	≥2	0.6109–0.7761

### 3.6 Typical case

A male patient admitted to our institution underwent comprehensive neuroimaging evaluation, which demonstrated extensive acute cerebral infarction involving the right frontal, temporal, parietal, and occipital lobes. On conventional MRI, the T1-weighted image showed a large hypointense area corresponding to the infarcted region, without definite identification of hemorrhagic components (Figure 1A). The T2-weighted image demonstrated extensive hyperintensity consistent with infarction, with internal focal hypointense areas of nonspecific appearance (Figure 1B). Diffusion-weighted imaging demonstrated extensive hyperintensity in the corresponding region, consistent with acute ischemic infarction (Figure 1C). On susceptibility-weighted imaging, multiple irregular patchy hypointense foci were observed within the infarcted area on the magnitude image, with corresponding phase alterations on the SWI phase image (Figures 1D, E, yellow arrows). Non-contrast CT showed subtle patchy hyperdensities in the affected region (Figure 1F, yellow arrows).

To further illustrate susceptibility-related imaging features observed on SWI in ischemic stroke, additional representative cases from different patients were included, demonstrating prominent vessel sign (PVS), susceptibility vessel sign (SVS), cerebral microbleeds (CMBs), and hemorrhagic transformation (HI) (Figures 2A–D).

## 4 Discussion

IS is one of the leading causes of death and disability worldwide, and its early diagnosis and prognostic evaluation are of great importance for clinical decision-making and rehabilitation management. However, currently used imaging modalities, such as DWI and MRA, although highly sensitive in the acute phase, remain limited in identifying small hemorrhages, thrombus composition, and blood–brain barrier disruption (18, 19). SWI, due to its high sensitivity to deoxyhemoglobin and hemosiderin, can simultaneously display venous return status, thrombus signals, and CMBs, and has therefore attracted increasing attention (20, 21). Against this background, the present study systematically compared SWI features between acute-phase and recovery-phase IS patients and analyzed their association with neurological outcomes, aiming to explore the clinical value of SWI in stage-specific diagnosis and prognostic prediction.

Our findings demonstrated that the positive rates of the PVS and HI, as well as the number of CMBs, were significantly higher in the recovery phase than in the acute phase, whereas the SVS showed no difference between groups. This indicates that SWI can capture pathological changes at different stages of disease progression. An increased PVS reflects reduced venous oxygen saturation and impaired venous drainage around the lesion, suggesting persistent ischemia (21); HI was more frequent during the recovery phase, consistent with clinical patterns of reperfusion-associated blood-brain barrier disruption and hemorrhagic transformation; and the marked increase in CMBs indicates that small-vessel pathology and microcirculatory disturbances become more prominent during the recovery phase. In line with previous reports (22), PVS has been regarded as an imaging marker of cerebral hypo perfusion, HI is closely associated with reperfusion injury, and CMBs have been repeatedly confirmed in cohort studies to strongly correlate with poor stroke outcomes (23). By systematically comparing these markers within the same population, our study strengthens their value in stage differentiation.

Neurological function assessments also showed significant differences. NIHSS scores at admission and discharge were markedly higher in the acute-phase group, while mRS scores at three months were higher and Barthel Index scores were lower compared to the recovery-phase group. These findings align with the natural disease course and further support the clinical relevance of SWI markers. Correlation analyses revealed that PVS, SVS, HI, and cerebral microbleed number were all positively associated with NIHSS and mRS scores but negatively associated with Barthel Index scores, suggesting a close relationship between SWI features and functional impairment. Notably, cerebral microbleed count exhibited the strongest correlation, indicating that it not only reflects acute injury burden but also imposes long-term limitations on functional recovery. Previous literature has similarly demonstrated that CMBs, as markers of small vessel disease, are strongly linked to stroke recurrence and cognitive impairment, which is highly consistent with our findings (24, 25).

ROC analysis further revealed the prognostic value of SWI parameters. Although PVS, SVS, and HI all showed some predictive ability, their performance was limited, whereas cerebral microbleed count had the highest AUC, outperforming the other markers. This suggests that cerebral microbleed burden may represent the most valuable imaging risk biomarker in clinical practice. Multiple previous studies have also reported that cerebral microbleed number is significantly associated with hemorrhagic transformation, stroke recurrence, and long-term disability risk (23, 26). Our study validates these findings across different stroke phases, providing strong evidence for incorporating CMBs into risk stratification frameworks. From a mechanistic perspective, each SWI marker relates to prognosis in distinct ways (21): PVS reflects oxygen metabolism disturbance and venous deoxyhemoglobin accumulation in ischemic regions, suggesting hypo perfusion (7); SVS reveals the deoxyhemoglobin component within thrombi, indicating occlusion of the culprit vessel (27); HI reflects blood–brain barrier disruption during reperfusion injury, often worsening neurological outcomes; while CMBs arise from fragile small vessel walls, chronic hypertension, and underlying cerebral small vessel disease, representing cumulative vascular injury (28). These mechanisms explain the observed tight relationship between SWI findings, neurological deficits, and adverse outcomes, highlighting SWI's unique advantages in capturing the complex pathology of stroke.

The clinical implications of this study are threefold. First, SWI, as a supplementary sequence to routine MRI, entails no additional risk to patients, is simple to perform, and highly reproducible, thus offering excellent accessibility. Second, we demonstrated that SWI provides information on culprit vessels and thrombi in the acute phase, while in the recovery phase it reveals hemorrhagic transformation and CMBs, suggesting broad applicability across the entire disease course. Particularly, the predictive value of cerebral microbleed burden indicates its potential utility in risk stratification and individualized treatment decision-making. For instance, in patients with a high microbleed load, clinicians may need to adopt greater caution when considering anticoagulation or thrombolysis to minimize bleeding risk, while closer monitoring and follow-up during rehabilitation may improve long-term functional recovery and quality of life.

Nevertheless, several limitations must be acknowledged. First, this was a single-center study. Although our sample size was larger than that of earlier studies, it remains insufficient to fully eliminate selection bias, and the external generalizability of the results requires further validation in multicenter, large-scale prospective studies. Second, the follow-up period was limited to three months, which is inadequate to assess the ability of SWI to predict long-term recurrence and disability. Third, MRI, including SWI, was performed only at baseline, and no follow-up MRI examinations were obtained. Therefore, dynamic changes in SWI features over time could not be assessed, which may limit the interpretation of the temporal evolution of imaging markers in relation to clinical outcomes. Fourth, interpretation of SWI findings relied on radiologists' expertise and therefore carried some subjectivity; future studies should integrate artificial intelligence–based tools and quantitative analyses to enhance consistency. Fifth, this study did not combine SWI with other modalities such as perfusion imaging, functional MRI, or serum biomarkers, which may have underestimated the contribution of complex pathophysiological mechanisms to stroke outcomes.

In conclusion, this study systematically evaluated SWI features across different phases of IS and their associations with neurological function and prognosis. Our findings indicate that PVS, SVS, HI, and CMBs are closely related to functional impairment, with cerebral microbleed burden showing the strongest predictive value for poor outcome. These results not only provide new imaging evidence for stage-specific stroke diagnosis but also offer valuable insights for clinical risk stratification and individualized treatment. Future research should build upon multicenter, large-sample, and long-term follow-up data to further validate and optimize SWI-based markers, as well as explore their integration with other imaging and biological parameters, thereby advancing the precision and standardization of IS management.

## 5 Conclusion

SWI can sensitively capture pathological changes at different stages of IS, among which cerebral microbleed burden demonstrates the highest predictive value for unfavorable outcomes, providing an important reference for clinical risk stratification and individualized treatment.

## Data Availability

The original contributions presented in the study are included in the article/supplementary material, further inquiries can be directed to the corresponding author.
